# Supplementary material for: The Systems Biology Research Tool: evolvable open-source software
Source: BMC Syst Biol. 2008 Jun 29;2:55. doi: 10.1186/1752-0509-2-55 (PMC2446383; doi:10.1186/1752-0509-2-55)
Supplement: Additional file 1 — SBRT Archive. An archive of the current version of the Systems Biology Research Tool. [file 1752-0509-2-55-S1.zip › sbrt-1.4.0/doc/developers_guide/api/sbrt/shell/text/Formatter.html]

Formatter


|  |  |  |
| --- | --- | --- |
| **Overview** \| **Package** \| **Class** \| **Use** \| **Tree** \| **Deprecated** \| **Index** \| **Help** | |  |
| **PREV CLASS**   **NEXT CLASS** | **FRAMES**    **NO FRAMES**     **All Classes** |  |
| SUMMARY: NESTED \| FIELD \| CONSTR \| METHOD | DETAIL: FIELD \| CONSTR \| METHOD |  |


---


## sbrt.shell.text Interface Formatter<T>

**Type Parameters:**: `T` - the type of object this format applies to.

**All Known Subinterfaces:**: BooleanFormat, CatalystFormat, CatalystListFormat, CatalystSetFormat, ChemSpeciesFormat, CoeffFormat, CollectionFormat<E>, CorrResultsFormatter<T>, DirEdgeFormat<N>, DoubleFormat, FbaOptHeaderFormatter, IndexPairFormat<R,C>, IntegerFormat, IntervalFormat, LinearCombFormat<V>, LinearCombTermFormat<V>, LinkedHashSetFormat<E>, ListFormat<E>, LongFormat, MathExprFormat<E,V>, MatrixElementFormat<R,C,E>, PercentFormat, RxnNameExprFormat<E,S>, RxnNameFormat<T>, RxnNameListFormat, RxnNameOrExprFormat, RxnNameSetFormat, SimpleFormat<T>

**All Known Implementing Classes:**: AbstractLinkedHashSetFormat, AbstractListFormat, AmpersandSetFormat, BoolRelFormatV1, BoundedIntervalFormatV1, CatalystFormatV1, CatalystFormatV2, CatalystListFormatV1, CatalystSetFormatV1, CatalystVerifier, ChemSpeciesFormatV1, ChemSpeciesVerifier, CoeffFormatV1, CoeffFormatV2, CorrResFormatterV1, DirEdgeFormatV1, DoubleFormatV1, DoubleFormatV2, FbaOptHeaderFormatterV1, FileFormatFormat, IndexPairFormatV1, IntegerFormatV1, IntervalFormatV1, IrrevRxnFormatV1, IrrevRxnFormatV1, IrrevRxnFormatV2, LinCombTermFormatV1, LinCombTermFormatV2, LinearCombFormatV1, LinearCombFormatV2, LongFormatV1, MatrixElementFormatV1, NonEmptyStringFormat, PercentFormatVersion1, PipeListFormat, PipeSetFormat, RevRxnFormatV1, RevRxnFormatV2, RxnNameExprFormatV1, RxnNameFormatV1, RxnNameListFormatV1, RxnNameOrExprFormatV1, RxnNameSetFormatV1, RxnNameVerifier, SenseFormat, SimpleListFormat, SimpleSetFormat, StringFormat, TabListFormat, TabSetFormat, TrueFalseFormat, WhitespaceListFormat, WhitespaceSetFormat, YesNoFormat

---

``` public interface Formatter<T> ```

This interface is used to represent *formatters*.
A formatter is used to "convert" a single object into a
single formatted string.

**Author:**
:   This interface was written and documented by
    Jeremiah Wright while in the Wagner lab.

---

**Method Summary**

`<S extends T>
  
java.lang.String`

`format(S obj)`
  
          Returns a formatted string representation of the
provided object.

**Method Detail**

### format

```
<S extends T> java.lang.String format(S obj)
```

:   Returns a formatted string representation of the
    provided object.

    :   **Type Parameters:**: `S` - a type that extends the primary type of this format. **Parameters:**: `obj` - the object to be formatted. **Returns:**: a formatted string representation of the provided object.


---


|  |  |  |
| --- | --- | --- |
| **Overview** \| **Package** \| **Class** \| **Use** \| **Tree** \| **Deprecated** \| **Index** \| **Help** | |  |
| **PREV CLASS**   **NEXT CLASS** | **FRAMES**    **NO FRAMES**     **All Classes** |  |
| SUMMARY: NESTED \| FIELD \| CONSTR \| METHOD | DETAIL: FIELD \| CONSTR \| METHOD |  |


---
